# Hæmoptysis

**Published:** 1910-07-09

**Authors:** Herbert French

**Affiliations:** Assistant Physician to Guy's Hospital.


### Hospital Clinics.

### HÆMOPTYSIS.

By HERBERT FRENCH, M.A., M.D. (Oxon.), F.R.C.P. (Lond.), Assistant Physician to Guy's Hospital.

The following case may serve as an introduction to the differential diagnosis of hæmoptysis: The patient was a male, aged thirty-eight, who was admitted on January 1 for bringing up blood. His father had suffered from gout and died of heart disease; one of his brothers suffered from gout; there was no family history of tubercle. The patient was married and had three healthy children. Sixteen years ago he had a vesical calculus removed, and fifteen years ago he had an attack of typhoid fever. He had long been in the habit of taking about five or six pints of beer a day, and two or three "rums" before breakfast. For the last twelve months he had complained of a heavy sick feeling when he first got up in the morning. He had never had syphilis. Five years ago he had been to the hospital suffering from epistaxis; there had been no recurrence until recently, when about Christmas time he had another slight attack.

On January 1 he had been in bed about an hour when he felt a gurgling sensation in his throat, and was very alarmed to find his mouth full of blood; he thought he brought up about half a pint. He did not retch, neither was there any cough or choking sensation, but the blood simply seemed to well up into his throat. He at once came to the hospital, and was then coughing up bright red, frothy blood. He was admitted into a ward, and at once put to bed.

*Condition on Admission.*—He was a big stout man with a florid complexion, and with small dilated venules on his nose and cheeks. The sclerotics were very slightly jaundiced. There were no tophi. The fingers were not clubbed. There was no anasarca.

*Alimentary System.*—The tongue was not furred. The bowels were constipated. There were no hæmorrhoids. The abdomen was large and flabby. The edge of the liver could be felt two inches below the costal margin in the right nipple line; it was hard, and the surface as far as could be determined was smooth. The spleen could not be felt. The chest was very emphysematous. A few faint rhonchi were heard at the bases. There were no signs of phthisis.

The sputum consisted of a good deal of bright red blood-stained froth.

*Circulatory System.*—The pulse rate was 82; and the pulse was regular, of low tension, and the radial artery was not thickened. The cardiac impulse could not be seen or felt. The area of cardiac dulness was smaller than normal on account of the emphysema. In the fifth intercostal space, about half an inch inside the left nipple line, a distinct blowing systolic murmur could be heard; it could be traced outwards into the axilla for two inches, but better inwards for two inches towards the left border of the sternum. The second sound at the base of the heart was loud.

The urine was of specific gravity 1018; it contained a very slight trace of albumen; no blood. The urea amounted to 3 per cent.

The patient was allowed ice to suck, and he was kept flat on his back in bed on a milk diet. The following mixture was prescribed:—

| | |
|---|---|
| Tinct. opii. ... ... ... ... | ♏v. |
| Acid. sulph. aromatici ... ... ... | ♏x. |
| Syr. balsami tolutani ... ... ... | ʒj. |
| Aquam ad. ... ... ... ... | ℥j. |

Two tablespoonfuls to be taken every six hours.

January 3. He was still kept quite quiet, lying on his back, and was much better. He continued to spit up blood-stained sputum, more of a brownish than a red colour.

January 4. Better. Sputum still brownish in colour.

January 5. He was allowed to get up after tea.

January 8. He went out, feeling perfectly well. With the exception of emphysema nothing abnormal could be detected in the lungs. The sputum had been examined for tubercle bacilli, but with a negative result.

The first point of interest is that on two occasions—*i.e.* when seen by the house physician in the surgery and by the clerk in the out-patient department—he was thought to be suffering from hæmatemesis. The mistake was made on account of the alcoholic history, the alcoholic appearance of the patient, and the enlargement of his liver. A careful examination of the blood brought up and the symptoms associated with it showed later that in spite of the evidence of alcoholism and cirrhosis of the liver the blood had come from the lungs and not from the stomach.

When called to see a patient who has been bringing up blood, one's first consideration must be to determine whether it is a case of hæmoptysis or hæmatemesis. These two conditions can only be distinguished by carefully inquiring into the history of the symptoms and by an examination of the blood which has been brought up. When this patient was seen in the out-patient department he clearly stated that he had coughed up the blood, that he had not been sick, and had not even felt sick. He described the blood as being bright red in colour, frothy, liquid, and not in clots; and on examination it proved to correspond exactly to his description. From these data it was obvious that the condition was hæmoptysis, and not hæmatemesis. In hæmoptysis, therefore, the blood is coughed up, and a tickling or gurgling sensation in the throat, such as this patient experienced, is not an uncommon premonitory symptom; the blood may be mixed with sputa; the amount of blood may vary from a few streaks to a pint or more; it is bright red in colour and frothy; it is usually liquid when it is brought up, but may coagulate in the vessel which receives it; it is alkaline in reaction. If the hæmoptysis is very profuse, the blood may simply pour out of the mouth in a stream and prove rapidly fatal. After hæmoptysis has occurred the sputa may be blood-stained for several days; at first it is bright red in colour, but subsequently becomes darker and of a brownish tinge.

### Some Points in Diagnosis.

In hæmatemesis the blood is vomited, and may in consequence be mixed with particles of partly digested food. If the hæmorrhage is considerable, the blood may be bright red in colour and liquid; or clots of blood which are not at all, or only slightly, altered in colour may be noticed. If the hæmorrhage has been gradual and blood has remained for some little time in the stomach, as it commonly does in contact with the acid gastric juice, it becomes of a brownish colour and has been likened in appearance to coffee grounds. The cause of the alteration in the colour is the action of the gastric juice, which converts the oxyhæmoglobin into hæmatin. It is then acid in reaction. The following summary shows at a glance the chief differences between hæmoptysis and hæmatemesis:

| *Hæmoptysis.* | *Hæmatemesis.* |
|---|---|
| 1. The blood is coughed up. | 1. The blood is vomited. |
| 2. The blood is bright red in colour and frothy; it is never watery. | 2. The blood is dark in colour and not frothy. |
| 3. The blood may be mixed with sputa. | 3. The blood may be mixed with vomit. |
| 4. The blood is alkaline in reaction. | 4. The blood may be acid in reaction. |
| 5. There may be a previous history pointing to pulmonary or cardiac disease. | 5. There may be a previous history pointing to gastric or hepatic disease. |
| 6. Before the blood is coughed up there is often a sense of tickling or gurgling in the throat. | 6. Before the blood is vomited there may be a feeling of sickness, oppression in the epigastrium, faintness and giddiness. |
| 7. The motions afterwards are not altered unless the patient swallows a good deal of the blood, when they may be tarry. | 7. The motions are often tarry in appearance afterwards. |
| 8. Blood-stained sputa may be expectorated for several days after the attack. | 8. There are usually no sputa. |

The next point to bear in mind is that hæmatemesis may be caused by hæmoptysis. This is most likely to occur when the bleeding takes place in the night, the blood being swallowed as soon as it gets into the pharynx, the patient remaining asleep and quite unconscious of the occurrence. The frequency with which hæmoptysis occurs during the night, when the patient is at rest, is remarkable; in the majority of instances the incidence of bleeding excites coughing, and the patient wakes. The following case illustrates this point: About 8 A.M. one morning a woman, who had been admitted on the previous day for general weakness and a history of bringing up blood, brought up a rather large quantity of dark brownish vomit which resembled in appearance, and proved on examination to be, altered blood which had been in the stomach. Those who had already seen her had diagnosed hæmatemesis, and had suggested gastric ulcer or cirrhosis of the liver as its probable cause. A careful examination of the front of the chest—care being taken not to move the patient, and percussion as a part of the examination being omitted—revealed very definite signs of phthisis at the left apex; namely, flattening of the chest wall, deficiency of movement, bronchial breathing, and consonating râles. A subsequent examination of the sputa showed the presence of tubercle bacilli, and for some days after the hæmorrhage the sputa were streaked with blood. There were no signs of gastric ulcer, malignant disease of the stomach, or cirrhosis of the liver. The explanation of the blood being vomited was that hæmoptysis had occurred during sleep, and that the blood had been swallowed as soon as it reached the pharynx. In a small proportion of the cases of hæmatemesis caused by cirrhosis of the liver signs of phthisis may be found, as alcoholism not only causes cirrhosis of the liver but is an important predisposing cause of phthisis. Blood from the nose or nasopharynx and blood from the buccal cavity may also simulate true hæmoptysis in some cases.

### Causes of Hæmoptysis.

If, however, it is possible to arrive at the conclusion that one's patient is suffering from hæmoptysis, the next point to ascertain is the cause of this very important symptom in the particular case.

True hæmoptysis—*i.e.* hæmorrhage from the lungs, bronchi, trachea, or larynx—must be distinguished first of all from spurious hæmoptysis, by which is meant the spitting of blood derived from the nose, mouth, or pharynx. For instance: Epistaxis, ulceration of the pharynx, stomatitis, malingering (sucking the gums), etc. A careful examination of the nose, mouth, gums, and pharynx will generally suffice to distinguish this condition, and, further, the blood is apt to be watery, mixed with saliva, and non-aerated. There was no evidence of any morbid condition affecting the above-mentioned parts in the patient under consideration.

Phthisis is by far the commonest cause of hæmoptysis. The latter may be the very first sign of the disease, or may be the last, the hæmorrhage in the former circumstances being little more than streaks, whilst in the latter it may be so severe as to cause the patient's death. The amount of blood brought up is very variable. In advanced stages of phthisis the diagnosis is not difficult. The wasting and flattening of the chest wall, deficient movement, increased tactile vocal fremitus, bronchial breathing, consonating râles, bronchophony and pectoriloquy at one apex, with signs of less advanced disease at the other apex, are distinctive; examination of the sputum showing the presence of tubercle bacilli, and perhaps elastic fibres, is conclusive. As above stated, hæmoptysis may be the first evidence of phthisis; the diagnosis is then difficult, as the physical examination may not reveal any abnormal signs. Tubercle bacilli may be found in the sputa, however, at this stage, so that a careful examination of even the most insignificant amount of sputum must always be made before a definite or reliable opinion as to the cause of the hæmoptysis can be given. With the exception of emphysema there was no evidence of disease of the lungs in the patient under discussion, no tubercle bacilli could be found in his sputum, nor had he suffered from cough or wasting. There was evidence, however, of a definite cardiac lesion.

It is a very rare condition to find phthisis associated with chronic valvular disease of the heart, unless the latter is of congenital origin. The nature of the actual cardiac lesion in this case will be discussed presently. In phthisis the actual cause of the hæmoptysis is not constant: it may be due to a variety of different conditions:—

1. In the early stages of the disease it may be the result of an inflammatory hyperæmia, with rupture of the capillaries; the amount of blood expectorated is then usually small and it may only amount to streaking of the sputum.

2. The small vessels may actually become inflamed and softened or become directly invaded by tuberculous changes, and in consequence rupture if any extra strain is suddenly put upon them; for instance, during attacks of coughing. This condition may lead to a more profuse hæmoptysis, even quite early in the course of the disease.

3. When the malady is more advanced the caseation and the breaking down of the lung tissue may lead to the tearing across of one of the larger branches of the pulmonary artery. This change does not always lead to hæmorrhage, as the lumen of the affected vessel may previously have become blocked by a thrombus as a result of the adjacent changes in the lung. Fortunately the vessels generally become thrombosed first, but when they happen not to have done so the rupture may cause a very profuse and, very likely, fatal hæmorrhage.

4. The most important cause is the rupture of aneurysms of branches of the pulmonary artery in cavities in the lung. These aneurysms may be saccular or fusiform in shape, and they may vary in size from that of a pin's head to that of a pigeon's egg. They are formed as follows: A cavity in the lung is produced by the breaking down of the areas of caseation. In this process branches of the pulmonary vessels may be exposed on the walls of the cavity which leads to a weakening of the vessel wall on the side bordering the cavity, on account of the loss of support entailed by the destruction of the lung tissue. The vessel wall may also be softened as a result of external softening from periarteritis, and the weakened patch in the wall of the vessel may yield to the blood pressure, expand and form a small saccular aneurysm which bulges into the cavity. Occasionally a vessel will be seen stretching right across a cavity—a so-called "bridle"—such a vessel will have lost all surrounding support and may therefore dilate in all directions and thus form a fusiform aneurysm. The rupture of such an aneurysm will cause a very sudden and profuse hæmorrhage which may rapidly be fatal. Most museums have specimens to show this.

*(To be continued.)*

